# Coupled hydro-mechanical processes in rainfall-triggered mudstone landslides revealed by 3D laser scanning and model testing

**DOI:** 10.1371/journal.pone.0345165

**Published:** 2026-04-30

**Authors:** Chi Li, Ke Wang, Shuanhu Li, Yu Gao, Yang Cao

**Affiliations:** 1 College of Civil Engineering, Inner Mongolia University of Technology, Hohhot, China; 2 Key Laboratory of Geological Hazards and Geotechnical Engineering Defense in Sandy and Drought Regions at Universities of Inner Mongolia Autonomous Region, Inner Mongolia University of Technology, Hohhot, China; 3 College of Resources and Environmental Engineering, Inner Mongolia University of Technology, Hohhot, China; China Construction Fourth Engineering Division Corp. Ltd, CHINA

## Abstract

To elucidate the hydro-mechanical evolution of rainfall-triggered loess-mudstone interface landslides and improve monitoring and early warning, we conducted a large-scale indoor physical model test under artificial rainfall conditions. The model was instrumented with pore-water pressure and earth pressure sensors, as well as terrestrial laser scanning (TLS) for spatially continuous, hourly displacement mapping. XRF/XRD and mechanical tests were adopted to quantify the water-induced softening characteristics of mudstone.The results show a power-law increase in water absorption and rapid strength degradation, with the unconfined compressive strength decreasing from 4.90 MPa to 0.82 MPa within 3 h. Rainfall promotes the formation of an interfacial water film and argillation of mudstone, which weakens inter-particle bonding and significantly reduces the interface shear strength, representing the key trigger for sliding. Pore-water pressure evolves quasi-synchronously with rainfall but with a slight lag, exhibiting a three-stage pattern: stable – accelerated rise – rapid decline. TLS captured deformation precursors at the crest and slope surface at approximately 1560 min, providing a 140-min lead time over sensor-detected anomalies (approximately 1700 min).TLS-derived displacement fields cross-validate with pressure-based indicators to characterize progressive destabilization, which culminates in failure under continuous rainfall. This study clarifies the water-film-controlled softening mechanism and demonstrates the superior early-warning sensitivity of TLS for interface-type landslides, providing a scientific basis for multi-index fusion monitoring and the formulation of refined early-warning thresholds.

## 1. Introduction

Rainfall-induced landslides are prevalent across the Chinese Loess Plateau and adjacent arid-semiarid regions. Among them, interface-type failures along the loess-mudstone contact are frequent, abrupt, and difficult to warn against in practice. Prior studies have advanced our understanding along two main lines. Mechanistically, coupled hydro-mechanical (HM) analyses in unsaturated soils elucidate how rainfall infiltration reduces suction and strength under varying hydrologic and stratigraphic conditions [1–5]. Experimentally, rainfall model tests capture spatiotemporal evolution of pore-water/earth pressure, deformation, and failure scenarios under controlled conditions [6–14]. In parallel, terrestrial laser scanning (TLS) has matured for high-resolution slope deformation monitoring and discontinuity characterization, and for 3D stability assessment when integrated with modelling [15–20]. Nevertheless, three critical gaps persist for interface-type landslides at loess-mudstone contacts:

Mechanism gap: While many studies interpret macro-scale pore-pressure-displacement relations, quantitative evidence linking water uptake, argillation (mudstone disintegration), interfacial water-film formation, and shear-strength degradation at the contact remains limited; material-structure integrated validation tailored to the loess-mudstone binary system is scarce [10,21,22].

Monitoring gap: TLS is predominantly used on natural slopes or coupled with numerical analysis [15,17,20,23]. Under controlled rainfall, truly co-located, co-scaled, and co-frequency observations that fuse surface “areal” displacement fields with in-slope pore/earth-pressure time series are rare, hindering a robust determination of displacement precursors, their lead time relative to pressure anomalies, and cross-validated criteria.

Early-warning gap: Threshold-based rainfall warnings are effective regionally [24–26], but for interface-controlled, rapidly failing slopes, there is a lack of repeatable physical evidence to derive fine-grained, multi-indicator thresholds that couple displacement and pressure metrics [8,12].

To address these gaps, we design a large-scale indoor physical model of a loess-mudstone interface under artificial rainfall, instrumented with multi-layer pore-water and earth-pressure sensors and complemented by hourly TLS for spatially continuous deformation mapping. Concurrent XRF/XRD and mechanical tests quantify water-induced softening of mudstone. Our objectives are to: (i) elucidate the rainfall-driven HM pathway of “interfacial water-film formation”, “argillation and softening” and “shear-strength degradation”; (ii) quantify the lead time of TLS-derived precursors over point sensors (approximately 140 min) and cross-validate these with the three-stage pore-pressure evolution; and (iii) provide a data-backed methodology for multi-indicator threshold construction tailored to interface-type landslides.

The distinctive contributions of this study are:

Targeted interface-mechanism verification: We focus on the loess-mudstone binary system and provide an evidence chain for the interfacial water-film-controlled, argillation-induced shear degradation, establishing water-mineral/structure-strength linkages via XRF/XRD and strength tests, beyond prior macro-scale correlations [10,21,22].

Multi-scale sensor-TLS data assimilation: Under controlled rainfall, we achieve co-frequency fusion of in-slope pressure time series and surface 3D displacement fields, delineate the coupling between the “stable-accelerated rise-rapid decline” pore-pressure pattern and surface deformation, and quantify a ~ 140 min early-warning advantage of TLS with reproducible timing and spatial referencing [8,15–17,27–30].

Transferable data and threshold framework: We release a cross-validated workflow for deriving synergistic displacement-pressure indicators and thresholds for interface-type landslides, complementing and refining regional rainfall-threshold approaches [12,20,24–26].

Reproducible experimental design: We detail the rainfall protocol, sensor ranges and layout, and TLS acquisition and registration workflow, facilitating replication and integration with 3D numerical modelling and machine-learning based prediction [1,17–20,23,31].

Overall, we provide mechanistic evidence for rainfall-triggered softening and failure initiation at loess-mudstone contacts and demonstrate the superior sensitivity of TLS for precursor detection and early warning, supplying a reproducible pathway for monitoring and threshold design in layered, interface-controlled slopes.

## 2. Experimental apparatus and methods

### 2.1. Experimental materials

**Fig 1:**
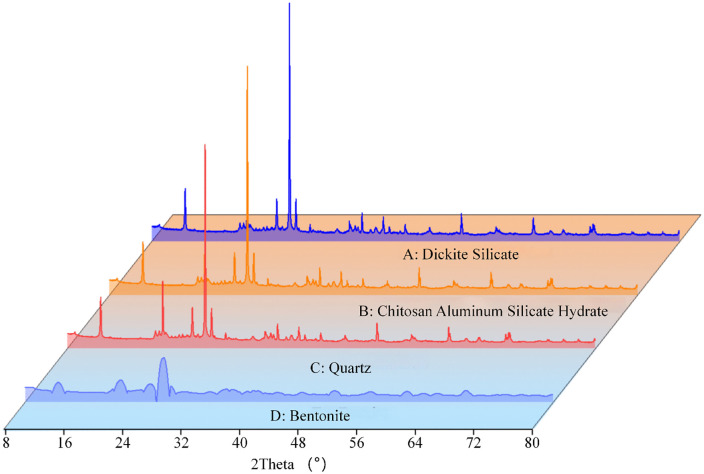
X-ray diffraction pattern of mudstone.

The mudstone samples used in this test were collected from central Inner Mongolia Autonomous Region. From a geological structural perspective, this area represents a transitional zone between the Shanxi platform anticline and the Inner Mongolia geosyncline. Geographically, the region is located at the northern margin of the Loess Plateau, with terrain features showing a decreasing trend from southeast to northwest. At the sampling site, the collected weathered mudstone samples exhibited typical brownish-red to purplish-red appearance. These samples primarily presented fragmented or blocky morphological characteristics due to long-term exposure to natural climatic and environmental factors. X-ray fluorescence spectrometry (XRF) was employed to precisely analyze the elemental composition and proportional content of the raw materials, with specific chemical compositions shown in Table 1. XRD was used for phase analysis of the mudstone, with the diffraction pattern (Fig 1). According to the XRD and XRF analysis results, the mineral composition of the mudstone primarily includes quartz (SiO_2_), calcium aluminum silicate hydrate (CaAl_2_Si_2_O_8_·4H_2_O), bauxite (Al_2_O_3_), and calcium silicate (Ca_2_SiO_4_).

**Table 1:** Chemical composition of mudstone.

Chemical Component	SiO_2_	Al_2_O_3_	Fe_2_O_3_	K_2_O	CaO	TiO_2_	MgO	Else
Content (%)	62.82	21.65	7.82	3.68	0.92	0.9	0.66	1.55

**Fig 2:**
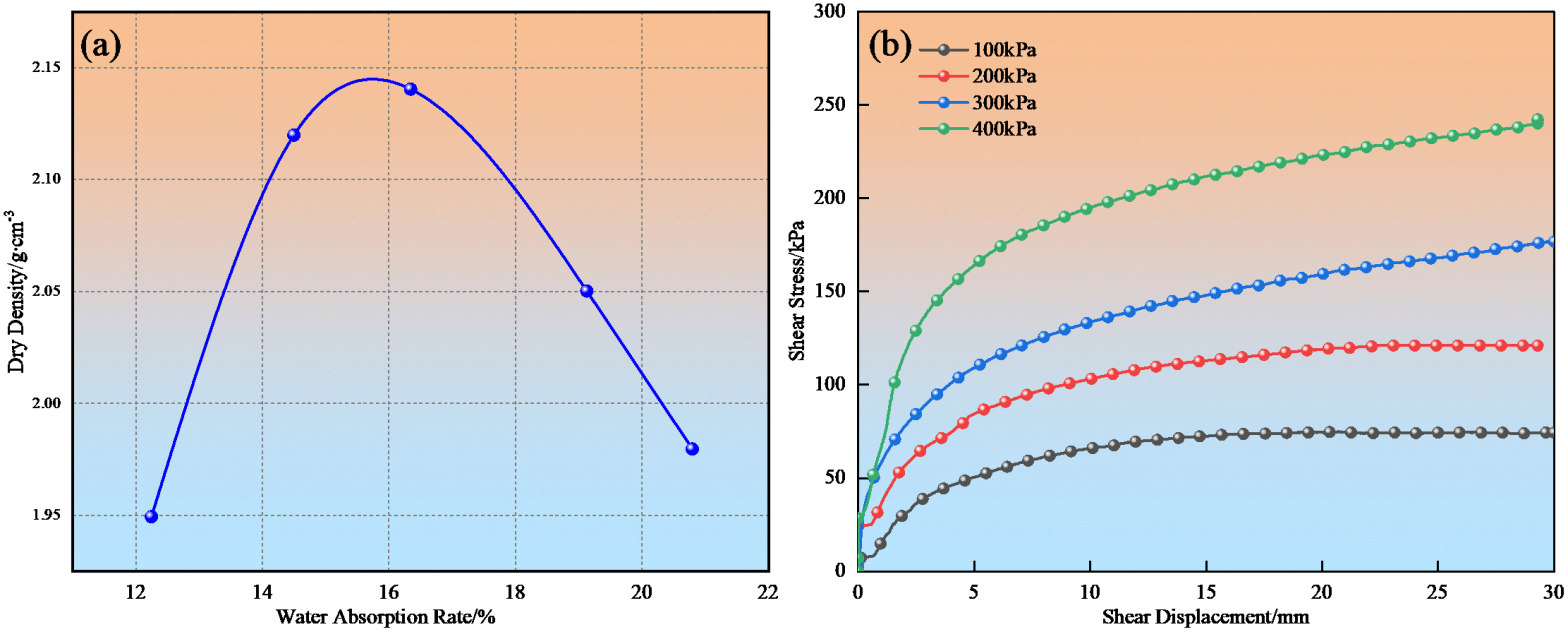
Compaction and direct shear test results: (a) Compaction test curve; (b) Shear stress-shear displacement relationship of mudstone under different interfacial water content conditions.

The compacted density of mudstone measured through indoor compaction tests was 1.66-1.79 g·cm-3, while that of loess was 1.4-1.5 g·cm-3. The mechanical properties of mudstone including cohesion, internal friction angle, and permeability coefficient are shown in Table 2, obtained through compaction tests (Fig 2a), direct shear tests (Fig 2b), and permeability tests. According to the plasticity chart in the “Highway Geotechnical Test Specifications” (JTG 3430−2020), the disintegrated mudstone particles used in this test are classified as low liquid limit clay, denoted as CL.

**Table 2:** Physical and mechanical parameters of experimental soils.

Material	Dry Density	Water Content %	Cohesion C/kPa	Internal Friction Angle Φ/(°)	Permeability Coefficient/ (cm/s)
Sliding mass	Mudstone 1.7 g·cm^-3^, Loess 1.45 g·cm^-3^	Loess 12%, Mudstone 15%	18.32kPa	27.26	5.26 × 10^−7^ cm/s

**Fig 3:**
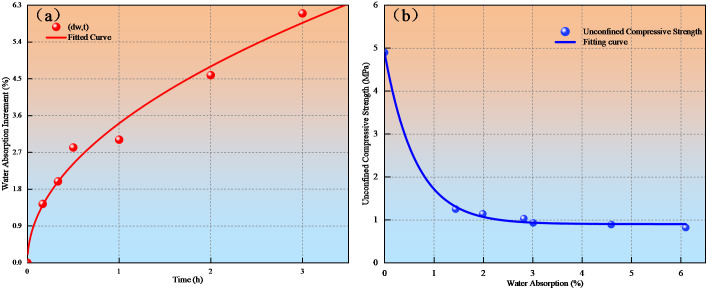
Mudstone water-induced softening test results: (a) Water absorption rate variation with time; (b) Unconfined compressive strength variation with water absorption rate.

**Fig 4:**
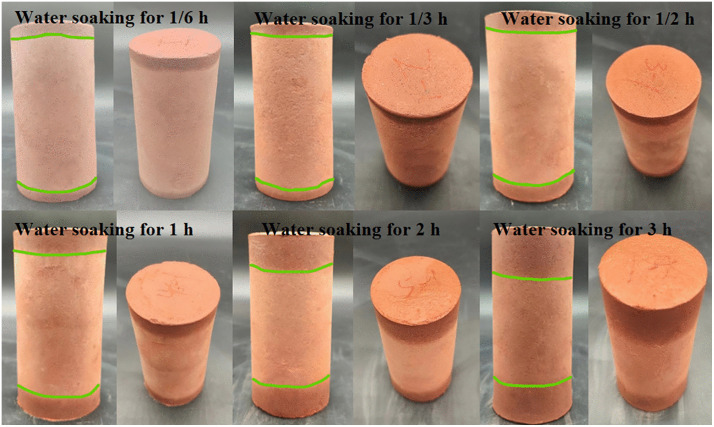
The diffusion phenomenon of mudstone after hygroscopicity.

Mudstone exhibits hygroscopic properties and easily dissolves and disintegrates when exposed to water. By designing tests with different soaking durations (10 min, 20 min, 30 min, 60 min, 120 min, and 3180 min), the results showed that water absorption increments after soaking for 1/6 h, 1/3 h, and 1/2 h were 1.44%, 1.99%, and 2.82%, respectively. Fitting the trend of mudstone water absorption increment over time revealed a distinct positive power function relationship, indicating rapid initial water absorption rates that gradually slowed and stabilized over time. The pattern of water absorption increment variation with time is shown in Fig 3(a). To study the influence of water-rock interaction on mudstone mechanical behavior, unconfined compressive strength tests with different soaking durations were conducted. The unconfined compressive strength of mudstone showed significant degradation with increasing water immersion time as shown in Fig 3(b). Mudstone visibly softened upon water absorption, with strength substantially decreasing; during initial water immersion, unconfined compressive strength rapidly reduced, transitioning from brittle fracture to plastic deformation. Mudstone exhibits hygroscopic properties and easily dissolves and disintegrates when exposed to water. By designing tests with different soaking durations (10 min, 20 min, 30 min, 60 min, 120 min, and 3180 min), the results showed that water absorption increments after soaking for 1/6 h, 1/3 h, and 1/2 h were 1.44%, 1.99%, and 2.82%, respectively. Fitting the trend of mudstone water absorption increment over time revealed a distinct positive power function relationship, indicating rapid initial water absorption rates that gradually slowed and stabilized over time. Variation in water absorption increment with time (Fig 3a). To study the influence of water-rock interaction on mudstone mechanical behavior, unconfined compressive strength tests with different soaking durations were conducted. The unconfined compressive strength of mudstone showed significant degradation with increasing water immersion time (Fig 3b). Mudstone visibly softened upon water absorption, with strength substantially decreasing; during initial water immersion, unconfined compressive strength rapidly reduced, transitioning from brittle fracture to plastic deformation. Water influence rapidly weakened the internal binding forces of mudstone, gradually transforming it from a hard state to one capable of plastic deformation (Fig 4). This plastic structure and softened state make mudstone prone to forming sliding zones; the combined effect of interfacial water films and softened mudstone becomes a key factor in landslide formation.

### 2.2. Experimental design

In this test, undisturbed loess and weathered mudstone collected near Honghe Town, Qingshuihe County, Hohhot, Inner Mongolia Autonomous Region were used as raw materials to construct a loess-mudstone binary model for simulating rainfall-induced interface landslides. To be consistent with typical regional geological conditions and ensure operability, the interface was set as a smooth type according to previous studies, and the dip angle of the mudstone layer was set to 16° (within the commonly reported range of 10°-20° in the literature, while also considering the engineering experience that angles of 15°-30° are favorable to slope instability) [32–38]. The overall slope geometry was optimized based on preliminary small-scale model tests.

The experiment was carried out in a laboratory with controlled climatic conditions to eliminate interference from external natural factors such as rainfall, temperature variation, and humidity fluctuation, thereby guaranteeing the stability of soil preparation, layered compaction, and static curing. The environmental parameters were set as follows: ambient temperature controlled at 20 ± 2°C and relative humidity maintained at 60% ± 5%. These conditions ensured uniform moisture loss during the air-drying of loess and mudstone, prevented soil agglomeration and large deviations in water content caused by over‑exposure or high humidity, and provided a stable environment for adjusting the soils to their target water contents (12% for loess and 15% for mudstone), minimizing the influence of evaporation on water content control.

To achieve uniform and repeatable material conditions, the bulk soil samples were air-dried, crushed, and sieved through 1 mm and 2 mm standard screens for loess and mudstone, respectively, to remove weeds, concretions, and other impurities. The amount of water required was calculated based on the measured initial water content, and the soil was thoroughly mixed and re-tested to achieve the target water content.

Layered compaction was adopted during slope preparation. Layer marks were drawn every 10 cm on the inner wall of the model box. Each layer was compacted manually with a wooden hammer, and the dry density was strictly controlled to ensure uniform density. The mass of soil required for each layer was calculated according to layer volume, target dry density, and water content. The soil was evenly spread and compacted to the marked level to meet the designed compaction degree. The surface of each layer was slightly scarified to improve interlayer bonding. To form an ideal weakened‑lubricated control surface, the mudstone layer was polished after compaction and slope trimming to reduce roughness and achieve the expected friction coefficient, followed by the placement and shaping of the loess layer. After construction, the slope was covered with plastic wrap and left standing for 1–2 days to allow the soil to reach initial stress equilibrium under nearly sealed conditions and prevent rapid moisture loss, providing a stable initial state for subsequent rainfall simulation [39–46].

The rainfall scenario was simulated using continuous intermittent heavy rainfall, as most slope failures in engineering practice are triggered by rainstorms. The total test duration was 3000 min. The rainfall intensity was designed to be no less than 2.1 mm per hour. Based on the model box dimensions (150 cm × 60 cm × 21 cm), the required hourly rainfall volume was determined to be 1.89 L. The total flow rate of the six nozzles was 1.32 L/min. According to the one‑hour rainfall interval, the theoretical duration for each rainfall event was 1.43 min, while the actual duration was set to 2 min to ensure sufficient coverage and uniformity, corresponding to a rainstorm-level intensity.

In this system, BW micro soil pressure sensors, BWK micro pore water pressure sensors (manufactured by Liyang Jincheng Testing Instrument Factory), and a 40-channel static digital resistance strain testing and analysis system were adopted to measure the pore water pressure and soil pressure at key internal points of the model, respectively.

A terrestrial 3D laser scanner was positioned 1.0 m directly in front of the open side of the model box to obtain an orthogonal view of the slope. Spherical targets (radius 0.0725 m) were deployed as control points to ensure registration accuracy and positional stability. Full scans were acquired immediately before rainfall, at predefined key stages during the experiment, and after slope failure to generate 3D point clouds. Rainfall duration, pore-water pressure, and other operating conditions were logged synchronously, enabling precise temporal alignment of the point clouds with the monitoring records [47–51].

### 2.3. Experimental apparatus

**Fig 5:**
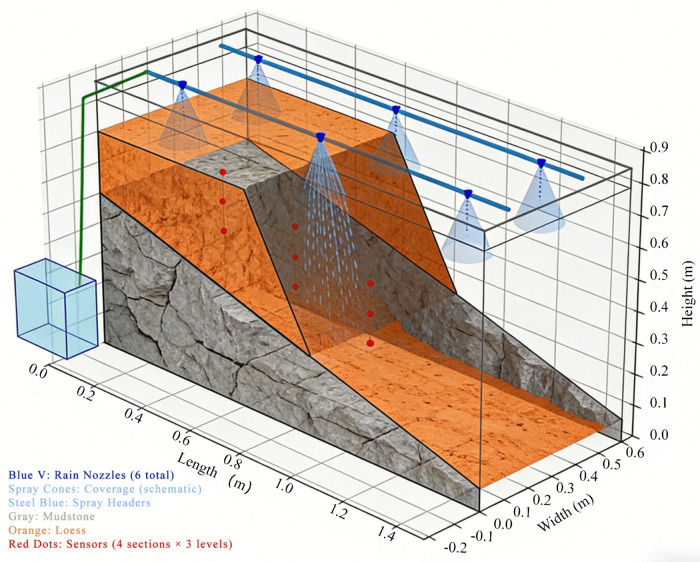
Schematic diagram of test apparatus and sensor measurement point layout.

To study the deformation evolution process of loess-mudstone interface landslides, this research employed large-scale indoor model testing combined with terrestrial laser scanning technology to investigate the effects of rainfall infiltration on mudstone interface softening and dynamic deformation of landslide bodies. The experimental setup primarily consisted of three components: test platform apparatus, artificial rainfall apparatus, and data acquisition system. The test model was scaled proportionally according to the structural characteristics of an actual landslide in a specific region. To analyze the stress field and seepage field variation characteristics of the landslide model test under rainfall conditions, four observation cross-sections were arranged at different positions, with a total of 12 earth pressure sensors and 12 pore water pressure sensors deployed across these sections to monitor changes in earth pressure and pore water pressure, respectively. One layer of sensors was embedded at the loess-mudstone contact interface, while the other two layers were embedded at the slope surface at vertical distances of 100 mm from the contact interface. According to the model’s top view, each observation point was equipped with one earth pressure sensor and one pore water pressure sensor, both positioned 20 mm to either side of the landslide body midline and embedded within the same horizontal plane. The test apparatus and sensor measurement point layout are shown schematically (Fig 5). High-speed laser scanning measurement technology was used to automatically and precisely scan the model area from left to right and top to bottom, obtaining overall and local deformation data for the target area.

The landslide model consists of three components: model box, secondary loess, and weathered mudstone. Based on landslide model similarity theory and similarity criteria, and referencing the structural characteristics of an actual regional landslide, the test model was proportionally scaled. The test utilized a model box with dimensions of 1.5 m × 0.6 m × 0.8 m (volume 0.72 m^3^), with one side of 0.6 m × 0.8 m constructed of transparent organic glass. During the preliminary design of indoor tests, to ensure test operability and simulation effectiveness based on multiple small-scale model test pre-studies, the dip angle of the mudstone layer was set to 16^°^. During slope filling, layered placement was adopted, with water content of test soils set at 12% for loess and 15% for mudstone. Mudstone was compacted to a density of 1.7 g·cm^-3^, while loess was compacted to 1.45 g·cm^-3^.

Referencing practical engineering applications where many slope failures are typically triggered by heavy rainfall, this test employed continuous intermittent rainfall simulation. Based on the dimensions and volume requirements of the test model box, the hourly precipitation needed to be 1.89 L to meet the established precipitation standards. During testing, the total water flow rate of six nozzles was 1.32 L/min. According to the requirement of one rainfall event per hour, each rainfall duration should be 1.43 min (actual rainfall duration was 2 min), corresponding to heavy rainfall intensity. The test parameters of the inclinometers, pore water pressure sensors, and earth pressure sensors used in the experiment are shown in Table 3.

**Table 3:** Sensor parameter indicators.

Name	Model	Range	Operating Temperature	Accuracy	Sensitivity Coefficient
Earth pressure sensor	BW miniature	60 kPa	−35- + 80°C	≤0.5%FS	K grade
Pore water sensor	BWK miniature	60 kPa	−35- + 80°C	≤0.5%FS	K: 2.00
Strain test analysis system	YBY-4010 type	±19999 με	–	Uncertainty: ≤ 0.5% ± 3 με	–

### 2.4. Experimental sensor arrangement

**Fig 6:**
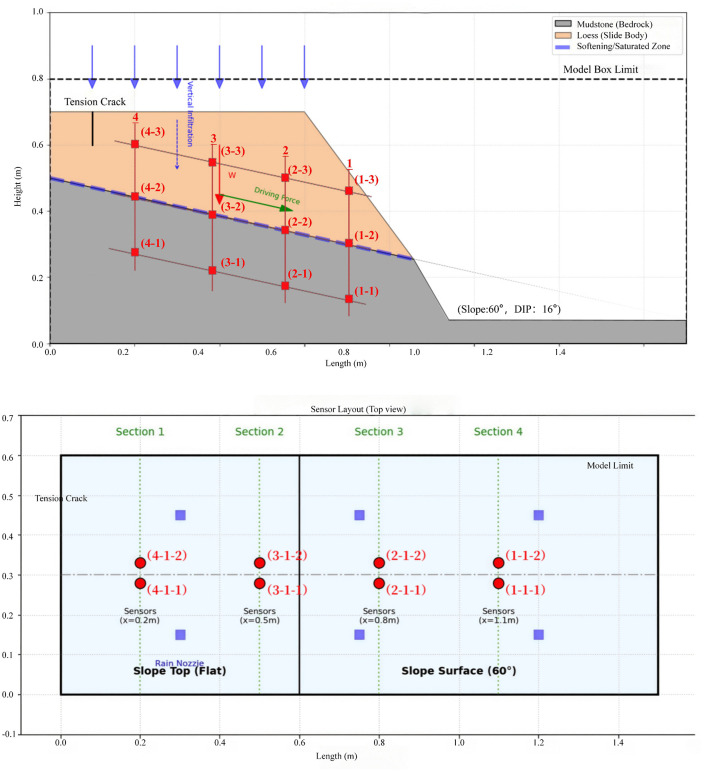
Measurement point layout diagram.

This test employed a binary conceptual model of “loess-mudstone”. Based on statistical survey data and to ensure the test could adequately simulate natural landslide occurrence conditions and achieve ideal test effects, the slope angle was set to 60°, the slope crest length designed as 60 cm, the rear edge height of the loess layer as 20 cm, and the rear edge height of the mudstone layer as 50 cm. To analyze the stress field and seepage field variation characteristics of the landslide model test under rainfall conditions, four observation cross-sections were arranged at different positions, with a total of 12 earth pressure sensors and 12 pore water pressure sensors deployed to monitor changes in earth pressure and pore water pressure, respectively. The loess layer had four observation points (1−1 to –4−1), the loess-mudstone interface had four observation points (1−2 to –4−2), and the mudstone interface had four observation points (1−3 to –4−3), with each interface having equally spaced horizontal observation points (Fig 6). Each observation point was equipped with both an earth pressure sensor and a pore water pressure sensor. High-definition cameras were used to photograph slope surface morphological changes at 20-minute intervals, while terrestrial laser scanners were used to scan the slope surface and generate three-dimensional topographic point cloud maps at 60-minute intervals. The rainfall process continued for a total of 3000 minutes.

To analyze the stress field and seepage field variation characteristics of the landslide model test under rainfall conditions, a total of 12 earth pressure sensors and 12 pore water pressure sensors were deployed, buried in three layers. One layer of sensors was embedded at the loess-mudstone contact interface, while the other two layers were embedded at the slope surface at vertical distances of 100 mm from the contact interface. According to the model’s top view, each observation point was equipped with one earth pressure sensor and one pore water pressure sensor, both positioned 20 mm to either side of the landslide body midline and embedded within the same horizontal plane. The pore water pressure sensors and earth pressure sensors were distributed across three interfaces, with four observation sections from slope crest to toe, respectively monitoring earth pressure and pore water pressure changes. All monitoring equipment on the model was networked to record displacement, pore water pressure, and earth pressure variations at different landslide positions during the failure process. After the soil mass stabilized under gravitational forces, and readings remained unchanged for 12 hours, the soil mass was considered to have reached a stable state. Subsequently, simulated rainfall began according to the predetermined rainfall scheme.

Terrestrial laser scanning technology was adopted for deformation monitoring of the landslide model test, and a FARO Focus Premium 350 terrestrial laser scanner was used as the core measurement instrument, whose main performance indicators are presented in Table 4. SCENE software, developed by FARO Corporation, was employed for the entire point cloud data processing process. To achieve high-precision alignment of multi-view point cloud data acquired at different test stages, a two-step registration strategy combining spherical target-based coarse registration and Iterative Closest Point (ICP) fine registration was adopted. Specifically, spherical targets with a radius of 0.0725 m were fixed at stable positions around the model box and served as common control points; by identifying and matching the three-dimensional coordinates of the same target in different scan stations, an initial transformation matrix was calculated to roughly unify all point clouds into a single coordinate system, laying a reliable foundation for subsequent fine registration. On the basis of coarse registration, the ICP algorithm was used for further precision improvement with the following optimized parameters: reference point cloud was the point cloud in the initial stable state obtained before rainfall, nearest neighbor search method adopted the k-d tree algorithm, maximum number of iterations was set to 100, convergence threshold was 1 × 10^−6^ m, maximum allowable distance for matching point pairs was 5 mm, and weighting mode was uniform weighting [52–57]. The ICP algorithm iteratively updated the rotation and translation matrices to minimize the mean square error of Euclidean distances between corresponding point pairs, and after the registration process was completed, the overall registration error was controlled within 0.1 mm, which fully satisfied the accuracy requirements for quantitative analysis of slope deformation during the test [58–60].

**Table 4:** FARO focus premium 350 scanner performance indicators.

Performance Indicator	Parameter
Visual range	614 m (maximum 0.5 Mpt/sec)
	307 m (maximum 1 Mpt/sec)
	153 m (maximum 2 Mpt/sec)
Scanning distance	White: 0.5-350 m
	Dark gray: 0.5-150 m
	Black: 0.5-50 m
3D accuracy	2 mm@10 m, 3.5 mm@25 m
Distance measurement error	±1 mm
Angular accuracy	19 arcsec
Color resolution	Color resolution up to 266 MPX
Field of view	300° vertical/ 360° horizontal
Maximum scanning speed	97 Hz (vertical)
Laser class	Class 1 laser
GNSS	Integrated GPS and GLONASS

## 3. Results

### 3.1. Slope failure description

At 2400 minutes, rainfall water continued to infiltrate, significantly increasing moisture content within the slope body. Obvious cracks appeared at the slope crest, initially revealing the sliding mass. The deformation rate of the landslide noticeably accelerated during this stage, with instantaneous displacement occurring in the slope body, accompanied by the fifth interfacial sliding failure. Water infiltration and sliding mass loosening became the main driving factors for landslide acceleration during this stage.

Deterioration stage: By 2520 minutes, slope crest cracks further expanded, severely threatening the overall stability of the slope body, followed by the sixth sliding failure. Crack expansion and fissure deepening indicated that the landslide had entered a deterioration stage, with slope stability rapidly decreasing and the destabilization process intensifying further.

**Fig 7:**
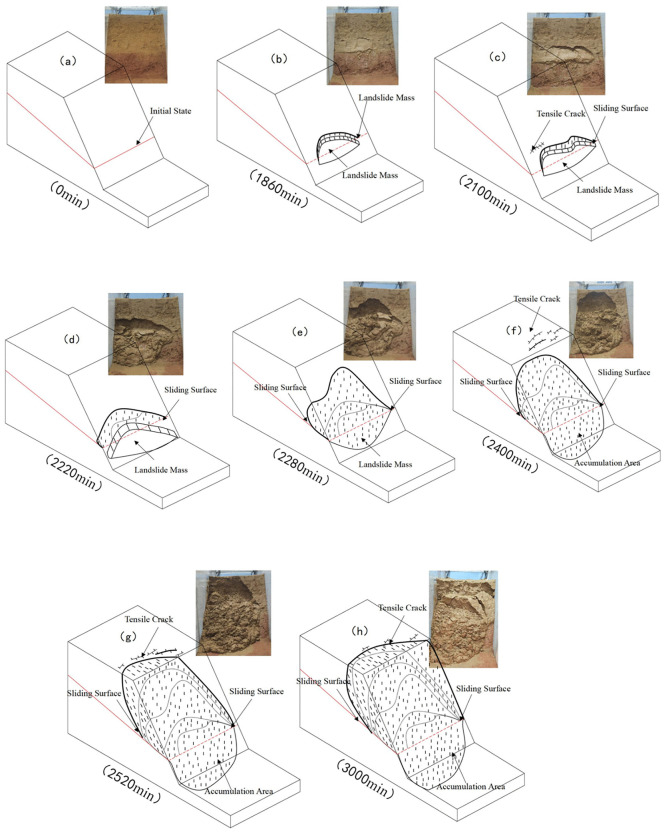
Failure mode demonstration diagram.

Complete destabilization: At 3000 minutes, slope crest cracks continued to expand, with the final sliding failure occurring along the interface, marking complete landslide destabilization. With continuous crack expansion, the slope body completely lost stability, and the sliding mass experienced large-scale sliding, completing the destabilization process (Fig 7).

### 3.2. Earth pressure and pore water pressure monitoring

**Fig 8:**
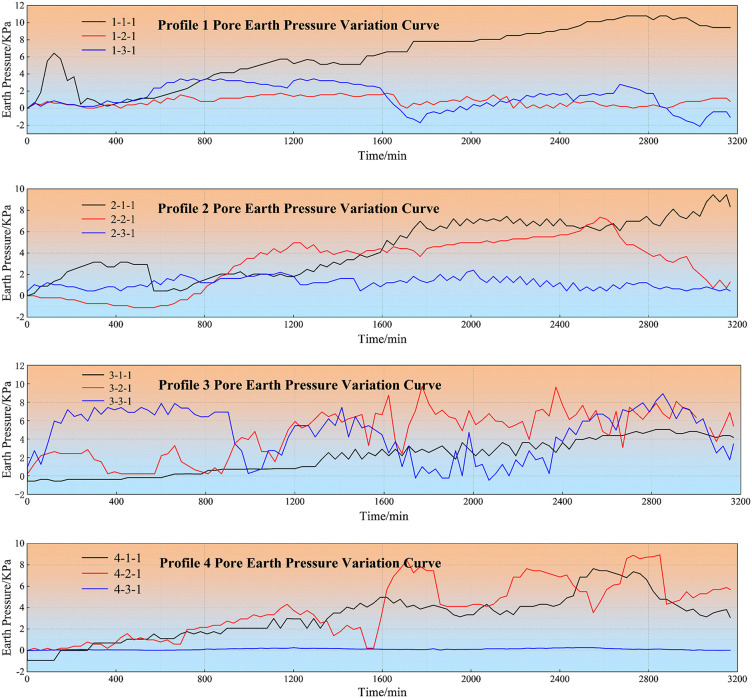
Earth pressure variation curve.

**Fig 9:**
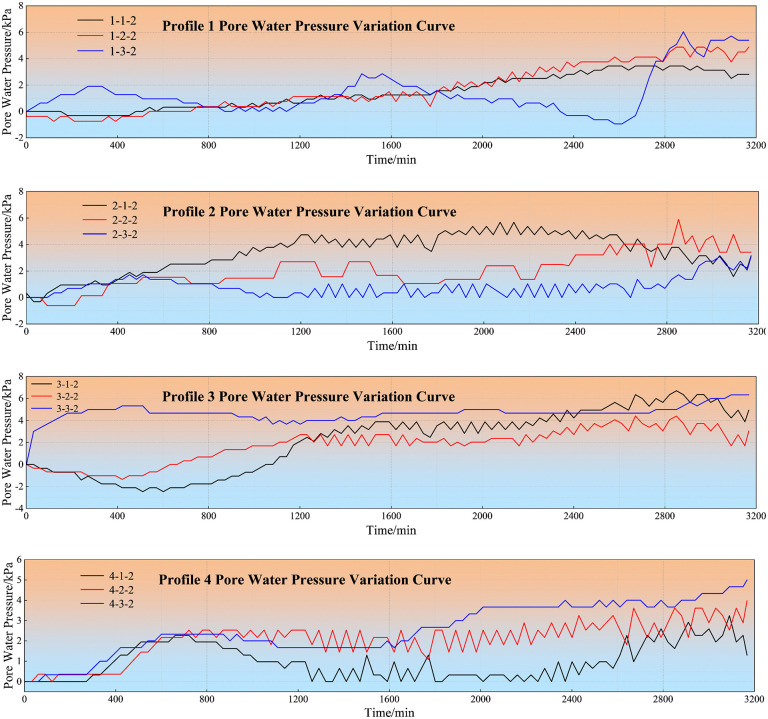
Pore water pressure variation curve.

This experiment monitored the variation curves of earth pressure and pore-water pressure in Fig 8 and Fig 9, from which pronounced patterns associated with landslide occurrence were identified. Furthermore, by analyzing the failure characteristics from unconfined compressive strength tests on mudstone specimens soaked for different durations, we elucidated the relationship between mudstone strength and water uptake, revealing the trend of mechanical property degradation after water absorption.

The monitoring data obtained from four pre-embedded pore water pressure sensors during the simulation test (Fig 9). From the figure, it can be observed that with gradual rainfall infiltration, both pore water pressure and earth pressure showed continuous increasing trends. Slope pore water pressure changes can be roughly divided into three parts: smooth section, accelerated rising section, and rapid declining section. According to the figure, during the initial rainfall period, pore water pressure at all four cross-section points remained around 0 kPa. Early on, due to small rainfall amounts, a certain amount of air existed in soil particle pores, preventing the formation of unobstructed permeation channels within the landslide body, thus pore water pressure within the soil mass did not change significantly. When rainfall duration reached approximately 1700 minutes, a sudden change occurred. Within this rainfall time span, slope toe deformation increased and stability decreased. This was because as rainfall continued to increase, soil particle pores gradually became filled with rainwater, transforming the soil mass from unsaturated to saturated state, with further increased permeability coefficient. Therefore, pore water pressure showed rapid increasing changes. This presaged landslide occurrence; actual observation found landslide occurrence at 1740 minutes, while the first interfacial sliding failure occurred at 1860 minutes. Due to differences in weak zone positions at the interface, earth pressure and pore water pressure changes exhibited certain differences. Research shows that pressure changes typically lag slightly behind the rainfall process in time, but generally synchronize with the rainfall process. At 2400 minutes of rainfall duration, the slope toe entered a deformation destabilization state, with internal soil pore water pressure showing a declining pattern. This time point corresponded with terrestrial laser scanning monitoring results showing landslide destabilization failure. The rapid decline in pore water pressure can be attributed to excessive rainfall content causing continuous reduction in soil shear strength until it became less than the applied shear stress, at which point the landslide exhibited destabilization sliding state, with internal pore water pressure being released, thus pore water pressure showed a declining trend.

As shown in Table 3, the results indicate that variations in pore water pressure are a direct manifestation of rainfall infiltration.. During rainfall, pore water pressure and earth pressure typically first decrease then increase, while during rainfall pauses they remain relatively stable. At landslide occurrence nodes, sensor data showed obvious abrupt changes. During early rainfall cessation periods, soil water pressure gradually stabilized, but rainfall increased soil unit weight, causing loess layer collapse settlement. Cracks near the interface expanded more rapidly, with water from upper soil masses accumulating along cracks toward the interface. Loess near the interface became oversaturated, reducing frictional resistance at the contact surface. At 1450 minutes of rainfall, pore water pressure suddenly increased significantly, with the loess layer at the interface ultimately experiencing overall sliding failure.

**Fig 10:**
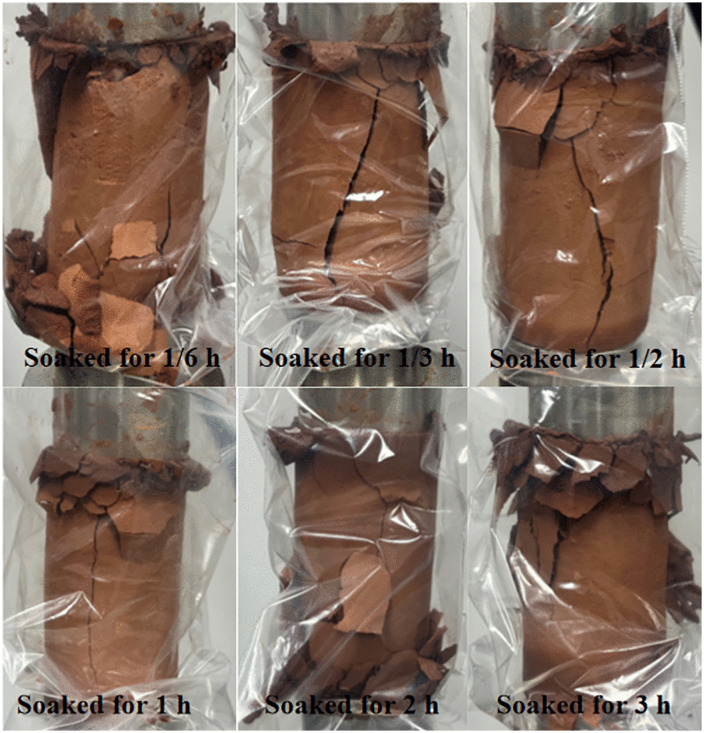
Unconfined compressive failure diagram of mudstone.

With a further increase in water soaking time, the strength of mudstone decreases significantly, and its failure mode is no longer sudden but rather characterized by gradual softening and deformation. As the deformation accumulates, the mudstone develops a large plastic deformation zone until eventual failure occurs. Driven by water infiltration, an interfacial water film forms on the mudstone surface, causing the internal particles to flow and rearrange, thus creating a softened mudstone layer. This process is referred to as argillation. When mudstone samples are subjected to unconfined compression tests under natural conditions, the failure is dominated by shear failure, with the positions of shear plane occurrence varying considerably (Fig 10). This phenomenon may be associated with the internal crack and fracture structures of the mudstone.The argillation process of mudstone not only impairs its mechanical properties but also alters the overall stability of the rock-soil mass. This plasticized and softened state renders the mudstone prone to sliding zone formation, where the combined action of the interfacial water film and the softened mudstone constitutes a key factor triggering landslides (Table 5).

**Table 5:** Results of mudstone water absorption test.

Water soaking time(h)	Unconfined Compressive Strength(MPa)
0	4.90
1/6	1.25
1/3	1.14
1/2	1.03
1	0.93
2	0.89
3	0.82

### 3.3. Terrestrial laser scanning

The total test duration was approximately 3120 minutes, with the final failure occurring at 3000 minutes. This study primarily focuses on analyzing slope surface displacement changes before landslide occurrence, especially before 1860 minutes.

**Fig 11:**
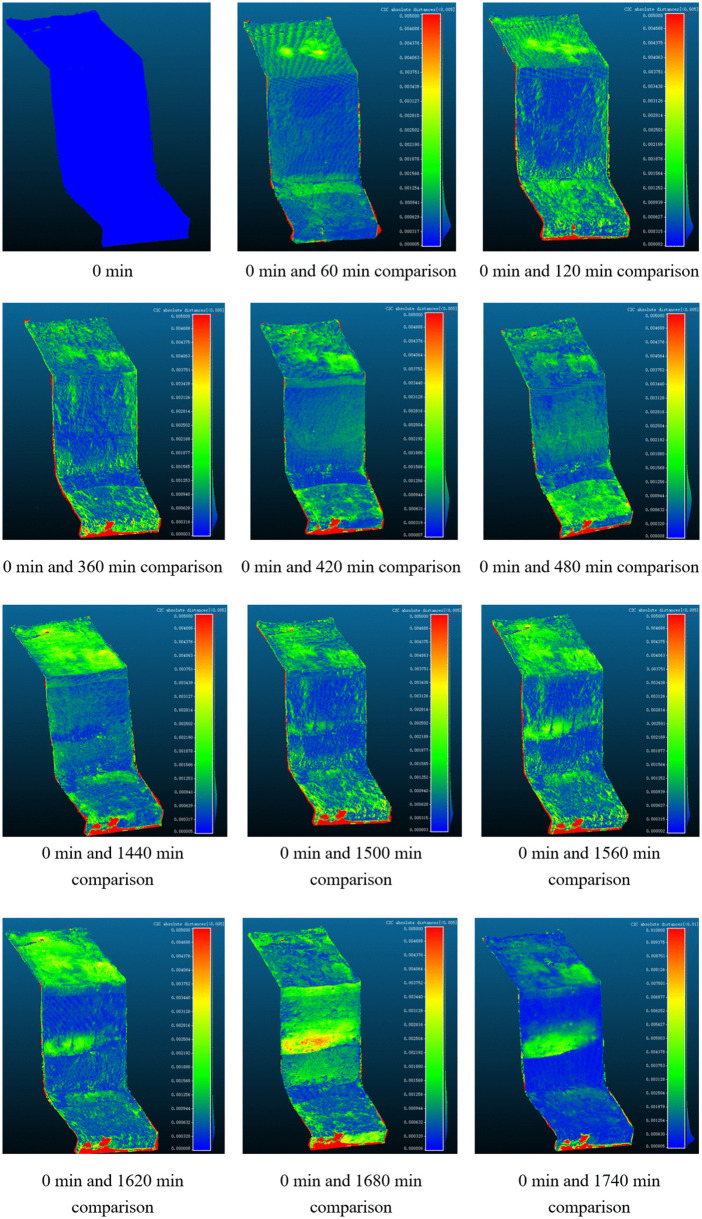
Displacement changes at various stages of test landslide.

By comparing scanning images, it can be observed that displacement changes at the slope crest and slope surface exhibited certain patterns (Fig 11). Each scanning interval was 60 minutes. With time progression, displacement at the slope surface and crest gradually increased, with slope surface displacement changes slightly lagging behind those at the slope crest.

From the slope crest perspective: During the 0–360 minute period, slope crest displacement changes primarily concentrated in the middle region of the slope crest. Since moisture had not yet fully penetrated to the loess-mudstone interface, slope crest displacement changes were minor. During initial rainfall penetration, moisture gradually infiltrated into the soil mass, increasing loess layer moisture content. However, due to relative stability of the slope surface layer, overall deformation was not significant. Displacement changes ranged between 0 to 0.0028 m.

During the 360–1620 minute period, rainfall amount continuously increased, with slope surface displacement changes gradually extending from the middle region to the entire slope crest area, indicating that loess layer moisture content gradually increased, causing widespread settlement of the loess layer. Displacement cloud map colors transitioned from green to light yellow, showing more significant displacement of slope crest soil under moisture effects. Displacement changes ranged between 0 to 0.0031 m, with this changing trend continuously expanding, proving the effects of continuous rainfall duration and moisture penetration on slope crest soil.

During the 1620–1860 minute period, maximum displacement changed, but overall displacement variation trends did not show significant fluctuations. Displacement cloud map changes still related to continuous rainfall penetration and moisture accumulation. Displacement changes during this stage were relatively stable, without major displacement variations. Displacement changes during this period further verified the gradual transition to stable state of slope crest soil.

From the slope surface perspective: During the 0–660 minute period, slope surface displacement changes primarily manifested as settlement of the loess layer surface. Affected by underlying layers, rainfall scouring effects reduced loose particles on the slope surface minimally. At this time, rainfall amount was relatively small, moisture penetration depth limited, and slope surface soil began absorbing water and gradually saturating. Due to relatively loose soil particles in the surface loess layer, after moisture penetration, soil particle binding forces weakened, causing local soil masses to experience minor subsidence or settlement phenomena. Displacement changes ranged between 0 to 0.0021 m.

During the 660–1680 minute period, with increasing rainfall amount, moisture gradually penetrated into slope surface soil and progressively reached the loess-mudstone interface. Slope surface displacement gradually increased, particularly during 720–760 minutes, when loess above the loess-mudstone interface began showing minimal sliding, with sliding distance of 0 to 0.0018 m. With time progression, sliding above the interface gradually intensified, especially at 1320 minutes, when the contact surface between loess layer and mudstone began showing sporadic failure, with sliding distance of 0.0021 m. With continuous precipitation, failure regions expanded from middle to both sides and slope crest, until at 1560 minutes, failure regions were clearly visible in the displacement cloud map, with displacement changes ranging between 0 to 0.0018 m.

Entering the 1680–1860 minute period, slope surface displacement changes further intensified. The displacement cloud map not only showed slope surface failure, but naked-eye observation also revealed local failure of the loess layer along the interface. Displacement changes showed expansion of sliding failure regions along the interface, with increased sliding distance indicating progressive intensification of slope instability. Displacement changes ranged between 0 to 0.01 m. Displacement changes during this stage reflected strong effects of precipitation and moisture penetration on slope surface soil.

Through analysis of displacement cloud maps, the occurrence process of loess-mudstone interface landslides can be clearly observed. According to displacement cloud map changes, initial failure indications became apparent around 1560 minutes. Slope surface displacement changes significantly increased, especially in the interface region between loess layer and mudstone, with significantly increased sliding distance presaging potential landslide risk. Sensor-monitored abrupt change time occurred around 1700 minutes, with a comparison showing approximately 140 minutes difference between sensor abrupt change time and failure time in displacement cloud maps. This discovery proves the important role of terrestrial laser scanning technology in landslide monitoring. Real-time analysis of displacement cloud maps enables early capture of minute soil movements, particularly during continuous precipitation when moisture penetrates soil layers causing settlement and gradual soil destabilization. Traditional sensor monitoring methods can only provide local data, while terrestrial laser scanning not only covers more comprehensive displacement information but also locks onto potential failure time points with higher precision, enabling more timely and accurate landslide early warning.

Further analysis reveals that the 140-minute early warning time results from terrestrial laser scanning’s ability to more precisely monitor minute soil displacement changes, with higher sensitivity to subtle changes at slope surfaces and crests. Traditional sensors may be affected by soil disturbance, installation positions, and data acquisition frequency, resulting in relatively lagged responses to displacement changes. This makes terrestrial laser scanning distinctly advantageous in monitoring and preventing loess-mudstone interface landslides, especially in engineering practice.

## 4. Conclusion

Based on actual engineering geological and boundary conditions, engineering geological simulation tests combined with artificial rainfall were conducted to explore water-rock interaction and moisture absorption-softening mechanisms at the sliding zone mudstone interface. Pore water pressure, earth pressure, and real-time dynamic deformation of the model landslide were monitored (via 3D laser scanning, TLS) to analyze landslide deformation and stability, with key conclusions drawn as follows:

(1) Sliding zone mudstone exhibits extremely poor permeability, resulting in slow internal water migration. Its physical properties (natural moisture content, density, liquid-plastic limit, compaction, and permeability) lead to a plasticized-softened state that facilitates sliding zone formation, where the combined effect of interfacial water film and softened mudstone is a key landslide-inducing factor.
(2) The “loess-mudstone” binary generalized model effectively simulates rainfall infiltration and mudstone softening. Water-induced interfacial water film on mudstone surfaces causes particle flow and rearrangement, forming a softened layer; mudstone argillation impairs its mechanical properties and rock-soil mass stability, with the synergistic effect of interfacial water film and softened mudstone being critical for landslide occurrence.
(3) TLS outperforms traditional sensor-based monitoring in loess-mudstone interface landslides with higher sensitivity and accuracy. TLS detected landslide precursor signs at ~1560 minutes, 140 minutes earlier than the ~ 1700 minutes abrupt change identified by sensors, confirming its capability to capture pre-landslide subtle soil displacement and provide valuable time for early disaster prevention.

## 5. Discussion

This study reveals the hydro‑mechanical coupling mechanism of rainfall‑induced loess-mudstone interface landslides, which overall follows the sequence “water-induced softening-interface lubrication - sliding-zone activation-accelerating failure.” Slope displacement and velocity exhibit marked temporal correlation with pore-water pressure (PWP): during the early infiltration stage, PWP rises slowly while surface displacement and velocity are essentially zero; as rainfall continues, water‑induced softening and interface lubrication markedly reduce interfacial shear strength, the displacement rate enters an acceleration phase, and shows a stable leading response relative to PWP (in this test, TLS leads PWP by about 140 min).

Placing TLS-derived surface displacement/velocity and PWP on a common time axis establishes a quantitative coupling that is crucial for accurately identifying the temporal evolution toward instability. TLS displacement captures the external deformation response of the slope, while PWP reflects the internal hydrologic evolution; cross-validating these two data streams mitigates the limitations of single-indicator analyses, and their concordant evolution supports the validity and reliability of the proposed failure mechanism.

The coupling between displacement and PWP may be influenced by soil heterogeneity and the distribution of the sliding zone. Future work should optimize the spatial deployment of TLS and PWP sensors to obtain higher-resolution spatiotemporal data, perform quantitative coupling on a unified time axis, and identify lead-lag relations, correlation strength, and time-varying windows, thereby deepening understanding of the hydro‑mechanical coupling in rainfall‑induced landslides.

## Supporting information

S1 File Mudstone water-induced softening test results. Data file containing the mudstone water-induced softening test results, including (a) water absorption rate variation with time and relevant test data, corresponding to the original file Fig 3.
(XLSX)

S2 File Earth pressure variation curve (1). Data file recording the first group of earth pressure variation curves during the test, corresponding to the original file Fig 8−1.
(XLSX)

S3 File Earth pressure variation curve (2). Data file recording the second group of earth pressure variation curves during the test, corresponding to the original file Fig 8−2.
(XLSX)

S4 File Earth pressure variation curve (3). Data file recording the third group of earth pressure variation curves during the test, corresponding to the original file Fig 8−3.
(XLSX)

S5 File Earth pressure variation curve (4). Data file recording the fourth group of earth pressure variation curves during the test, corresponding to the original file Fig 8−4.
(XLSX)

S6 File Pore water pressure variation curve. Data file recording the variation curve of pore water pressure during the rainfall-triggered mudstone landslide test, corresponding to the original file Fig 9−1.
(XLSX)

S7 File Pore water pressure variation curve (2). Data file recording the second group of pore water pressure variation curves during the test, corresponding to the original file Fig 9−2.
(XLSX)

S8 File Pore water pressure variation curve (3). Data file recording the third group of pore water pressure variation curves during the test, corresponding to the original file Fig 9−3.
(XLSX)

S9 File Pore water pressure variation curve (4). Data file recording the fourth group of pore water pressure variation curves during the test, corresponding to the original file Fig 9−4.
(XLSX)

S10 File Displacement changes at various stages of test landslide. Data file containing the displacement change data of each stage of the test landslide, obtained by 3D laser scanning, corresponding to the original file Scanning_data.
(XLSX)

S11 File Density and dry density of mudstone. Data file recording the density and dry density test data of mudstone samples used in the test, corresponding to the original file 密度与干密度.
(XLSX)

S12 File Direct shear test results of mudstone. Data file containing the direct shear test results of mudstone samples, providing basic mechanical parameters for the hydro-mechanical coupling model, corresponding to the original file the direct shear results of mudstone.
(XLSX)
